# Internet-delivered cognitive behavioral therapy for insomnia: The future of insomnia treatment with large language models

**DOI:** 10.1016/j.sleepx.2025.100157

**Published:** 2025-10-24

**Authors:** Chenxing Lin, Chen Cheng, Yu Xia, Ruoyun Wang, Luying Zhang, Lintong Han, Darong Hai, Chenyue Dai, Tianen Dai, Luhuan Cao, Xingyu Zhu, Hao Chen, Li Zeng, Chufan Ren, Dongren Yang, Yijun Wang, Meijia Zhang, Zefei Mo, Yinda Chen, Jianwei Shuai, Rongwen Yu, Yuming Peng

**Affiliations:** a Oujiang Laboratory (Zhejiang Lab for Regenerative Medicine, Vision, and Brain Health), 325000, China; b Wenzhou Institute, University of Chinese Academy of Sciences, Wenzhou, 325000, China; c The First School of Medicine, School of Information and Engineering, Wenzhou Medical University, Wenzhou, 325000, China; d School of Sports Science, Qufu Normal University, Qufu, Jining, 273165, China; e School of Nursing, Wenzhou Medical University, Wenzhou, 325000, China; f The Second Clinical Medical College of Wenzhou Medical University, Wenzhou, 325000, China; g School of Biomedical Engineering, School of Ophthalmology and Optometry, Eye Hospital, Wenzhou Medical University, Wenzhou, 325000, China; h School of Electrical and Information Engineering, Quzhou University, Quzhou, 324000, China; i Department of General Practice, Central Hospital of Karamay, Xinjiang, 834000, China

**Keywords:** Cognitive behavioral therapy for insomnia, Internet-delivered cognitive behavioral therapy for insomnia, Artificial intelligence, Large language models, Insomnia treatment

## Abstract

With the rise in the prevalence of insomnia, cognitive behavioral therapy for insomnia (CBT-I) has become an important non-pharmacological approach to the treatment of insomnia patients. Traditional CBT-I faces challenges such as low patient acceptance and long treatment cycles. To address these issues, Internet-delivered Cognitive Behavioral Therapy for Insomnia (eCBT-I) emerged as a digital treatment modality with greater flexibility, accessibility and cost-effectiveness. However, eCBT-I still faces issues such as digital literacy and patient engagement. With the development of artificial intelligence (AI) technology, especially the application of large language models (LLM), AI-driven CBT-I is becoming an important direction for future therapy. The LLM is able to provide personalized treatment recommendations based on patient feedback, improving treatment outcomes and patient engagement. This paper reviews the application status and challenges of CBT-I and eCBT-I, focuses on the potential and prospect of LLM in CBT-I therapy, and proposes future research directions, including multi-source data fusion and privacy protection issues, to promote the innovation and development of CBT-I.

## Highlights

• This review summarizes evidence on CBT-I/eCBT-I and AI, highlighting key gaps.
• ECBT-I provides similar effectiveness to in-clinic CBT-I while improving accessibility and reducing costs.
• LLMs enable personalized, adaptive CBT-I that enhances patient adherence and treatment outcomes.
• Multimodal data (e.g., wearables, e-diaries, EEG) help create customized sleep interventions.
• Incorporating human-in-the-loop and privacy-by-design principles ensures safe, personalized insomnia treatment.

## 1 Introduction

Insomnia is a prevalent sleep disorder characterized by difficulty initiating sleep within 30 min, frequent nocturnal awakenings, and reduced sleep quality, which often leads to daytime fatigue, impaired concentration, and mood disturbances [1,2]. According to the Diagnostic and Statistical Manual of Mental Disorders, Fifth Edition (DSM-5), a diagnosis of chronic insomnia is made when these symptoms occur at least three times per week for a period exceeding three months [3]. The consequences of insomnia extend beyond the physical health and cognitive impairment, substantially lowering quality of life and increasing the risk of anxiety and depression [4]. The development of insomnia is a multifaceted process that is influenced by a variety of factors [5,6]. Research has identified several potential causative factors, including cognitive biases, poor sleep habits, and chronic stress-induced over-activation of the autonomic nervous system. Cognitive bias may cause individuals to become overly preoccupied with sleep problems, thus exacerbating insomnia symptoms [7]. Additionally, unhealthy sleep behaviors, such as irregular work schedules or excessive reliance on electronic devices before bedtime, can disrupt normal sleep patterns [8]. Prolonged stress exposure can further impair autonomic function, worsening insomnia severity [9,10]. Current therapeutic interventions for insomnia encompassed non-pharmacological approaches, such as CBT, and pharmacological treatments, including benzodiazepines or melatonin, which were selected based on the patient's medical history and exhibited symptoms [11].

CBT is a psychotherapeutic approach that is designed to improve mental health problems by identifying and changing maladaptive cognitive and behavioral patterns. It is a short-term, structured, and problem-oriented approach [12]. Its central principle is that dysfunctional thinking patterns shape emotions and behaviors, and targeted cognitive restructuring can reduce distress and improve functioning. Typically short-term and goal-oriented, CBT involves collaboration between patient and therapist to set specific objectives and implement behavioral and cognitive strategies [13,14]. Through this combination of interventions, CBT effectively reduces patients' suffering and enhances their ability to cope with life's challenges.

CBT-I is a structured multimodal intervention specifically designed for insomnia that combines cognitive restructuring with behavioral interventions to improve sleep efficiency and quality [15]. In patients with insomnia without psychiatric co-morbidities, CBT-I has demonstrated significant efficacy in improving subjective insomnia severity, shortening sleep latency, decreasing the time of awakening after sleep onset, and alleviating daytime symptoms [[16], [17], [18]]. Its key components include cognitive therapy to address maladaptive sleep beliefs, sleep restriction to consolidate sleep, stimulus control to reinforce the bed–sleep association, relaxation training to reduce pre-sleep arousal, and sleep hygiene education to promote healthy routines [[19], [20], [21], [22], [23]]. CBT-I has received widespread recognition from the international medical community as the preferred treatment for insomnia, and its efficacy is supported by a substantial body of research [16].

In comparison with medication, CBT-I has long-term efficacy and not only significantly improves the subjective sleep quality of patients but also maintains its efficacy over a long period of time after the conclusion of the treatment with a low risk of relapse [16]. In contrast to sleep medications such as benzodiazepines, CBT-I does not lead to drug dependence or tolerance issues [24]. Furthermore, CBT-I has been shown to be effective not only for primary insomnia, but also for insomnia patients with co-occurring psychological problems, including anxiety, depression, and PTSD [14,25]. Research has demonstrated that CBT exhibits modest superiority over medication in enhancing sleep latency and improving sleep quality [[26], [27], [28]]. Moreover, the efficacy of CBT persists over time, while medication, although initially more effective, is susceptible to relapse following discontinuation [29]. Integrated therapy (CBT combined with medication) may offer additional advantages, though outcomes vary by patient. Increasingly, Internet-delivered CBT-I has also shown comparable efficacy, highlighting new opportunities for scalable care. Thus, treatment selection should weigh immediate and long-term outcomes, patient preference, accessibility, and delivery format, including whether CBT-I is combined or sequenced with pharmacotherapy [30].

This article reviews the application, challenges and future directions of CBT-I and eCBT-I in the treatment of sleep disorders. Firstly, the basic methods of CBT-I and its effect in the treatment of insomnia are introduced. Secondly, the challenges faced by traditional CBT-I, such as low patient acceptance and long treatment cycles, are discussed. The advantages of eCBT-I were then explored, particularly its potential in improving accessibility and reducing treatment costs. Finally, the application prospect of AI technology, especially large-scale language model in CBT-I is analyzed, and the challenges of privacy protection and technology optimization are raised.

## 2 Application and challenges of traditional CBT-I and eCBT-I

### 2.1 Challenges faced by traditional CBT-I

Insomnia is a prevalent and costly health problem, and many patients still do not receive timely or effective treatment [31]. CBT-I is now widely recognized as the treatment of choice for insomnia and has been incorporated into clinical practice guidelines by several international medical and sleep organizations [32,33]. However, despite its efficacy in treating chronic insomnia, its role in acute insomnia remains debated [24]. In cases of acute insomnia, medication is often considered a quick and effective option [16]. The combination of medication and CBT-I has been shown to rapidly alleviate acute insomnia symptoms; however, concerns regarding medication side effects and potential dependency issues persist. Therefore, how to balance the use of pharmacological treatment and CBT-I, particularly in finding the optimal balance between short-term effectiveness and long-term outcomes, remains an important issue of concern [34].

The implementation of CBT-I faces several barriers despite its status as the gold standard. Patient acceptance is often low due to limited awareness or confidence in CBT-I, leading to weak engagement [35]. A shortage of trained psychotherapists, especially in resource-limited regions, further constrains access [36,37]. The prolonged nature of the treatment period, a key component of CBT-I, can impose a significant burden on patients with demanding schedules, potentially compromising their adherence to the treatment and its overall effectiveness [35]. Concurrently, individual differences in the presentation and etiology of insomnia exist, thereby resulting in the efficacy of CBT-I varying from person to person. The efficacy of CBT-I may be suboptimal in patients with other concomitant health problems, necessitating individualized adjustments to the treatment regimen [38]. Overall, the diffusion of CBT-I must overcome low acceptance, high treatment burden, and heterogeneous patient responses to achieve broader implementation and stable efficacy.

### 2.2 The rise and advantages of eCBT-I

In recent years, with the advancement of technology, digital therapies, especially eCBT-I, have emerged as an innovative way to address these limitations [38]. eCBT-I, a non-pharmacological intervention based on traditional CBT, leverages Internet platforms, mobile apps, and other digital tools to overcome geographical barriers and limited service availability, enabling remote delivery and self-management [45]. Typically, eCBT-I consists of 8–12 patient-oriented and goal-oriented pre-designed sessions that are delivered via phone, mobile apps, or web platforms. Psychotherapist instruction can be delivered synchronously (e.g., by phone or videoconference) or asynchronously (e.g., by secure email or web-based communication platforms) [39].

Compared to traditional face-to-face CBT, eCBT-I has significant advantages. Its flexibility in content and delivery, lower entry barrier, reduced cost, and scalability make it highly cost-effective [39]. It is particularly suitable for resource-poor settings or time-constrained patients, as it significantly reduces the demand for direct therapist interaction. The spread of broadband networks and smartphones has further established eCBT-I as a standalone therapeutic option, contributing to advances in managing psychiatric disorders, including insomnia [40].

The efficacy of eCBT-I has been supported by multiple studies. For example, Nilofar Rajabi Majd et al. conducted a randomized controlled trial demonstrating improvements in sleep hygiene, quality, and disorder severity. Xu Yan et al. reported that eCBT-I effectively improved sleep across age groups while maintaining strong adherence [41,42]. A meta-analysis by Robert Zachariae et al. confirmed that eCBT-I significantly improves insomnia severity, sleep efficiency, sleep quality, latency, and total sleep time, achieving results comparable to face-to-face CBT-I and sustaining efficacy during follow-up (4–48 weeks) [43].

Beyond clinical outcomes, eCBT-I is also cost-effective. Claudia Buntrock et al. showed that it enhanced well-being, increased QALYs, and was cost-beneficial compared with wait-list control groups [44]. Taken together, current evidence indicates that eCBT-I not only alleviates insomnia symptoms but also improves adherence and reduces healthcare expenditures, providing a strong foundation for its wider clinical use.

### 2.3 Challenges for eCBT-I

However, the implementation of eCBT-I is encumbered by numerous challenges. Primarily, some patients may lack the requisite digital skills to utilize the platform effectively, thereby constraining its popularity [12]. Additionally, the absence of face-to-face interaction may result in patients not receiving prompt guidance when encountering challenges, thereby increasing the likelihood of treatment interruption [45]. The efficacy of eCBT-I is contingent on patient engagement; however, patients may encounter difficulties such as motivation or self-management challenges during the course of using the platform, which can substantially impact the treatment outcome [46]. The sustainability and stability of its therapeutic effects must be evaluated by further research [47]. Face-to-face treatment is still considered more effective when professional guidance and continuous support are required [48]. Therefore, how to improve patient engagement and adherence, as well as how to compensate for the lack of interpersonal support limitations in digital treatments, remains a key challenge in promoting the widespread use of eCBT-I.

Beyond technical barriers, adherence is one of the most persistent challenges in eCBT-I. Although its efficacy has been well demonstrated, dropout rates remain high, and many users fail to complete all modules or maintain regular sleep diaries. In 2016, Zachariae et al. conducted a systematic review and meta-analysis showing that eCBT-I can achieve outcomes comparable to face-to-face CBT-I, but only when adherence is sufficient; poor compliance substantially reduces treatment benefits [43]. In 2020, van der Zweerde and colleagues highlighted that low digital literacy, the lack of therapeutic alliance in unguided formats, competing life demands, and transient daytime sleepiness caused by sleep restriction therapy are all major factors undermining adherence [49]. In 2020, Baglioni and colleagues further suggested that introducing guided or blended CBT-I for high-risk populations can safeguard long-term efficacy and adherence [44]. Thus, adherence is not only critical for clinical effectiveness but also directly determines the long-term cost-effectiveness of eCBT-I.

## 3 Current status and applications of AI in CBT-I treatment for sleep disorders

### 3.1 The development trends of AI-driven CBT-I

**Fig. 1:**
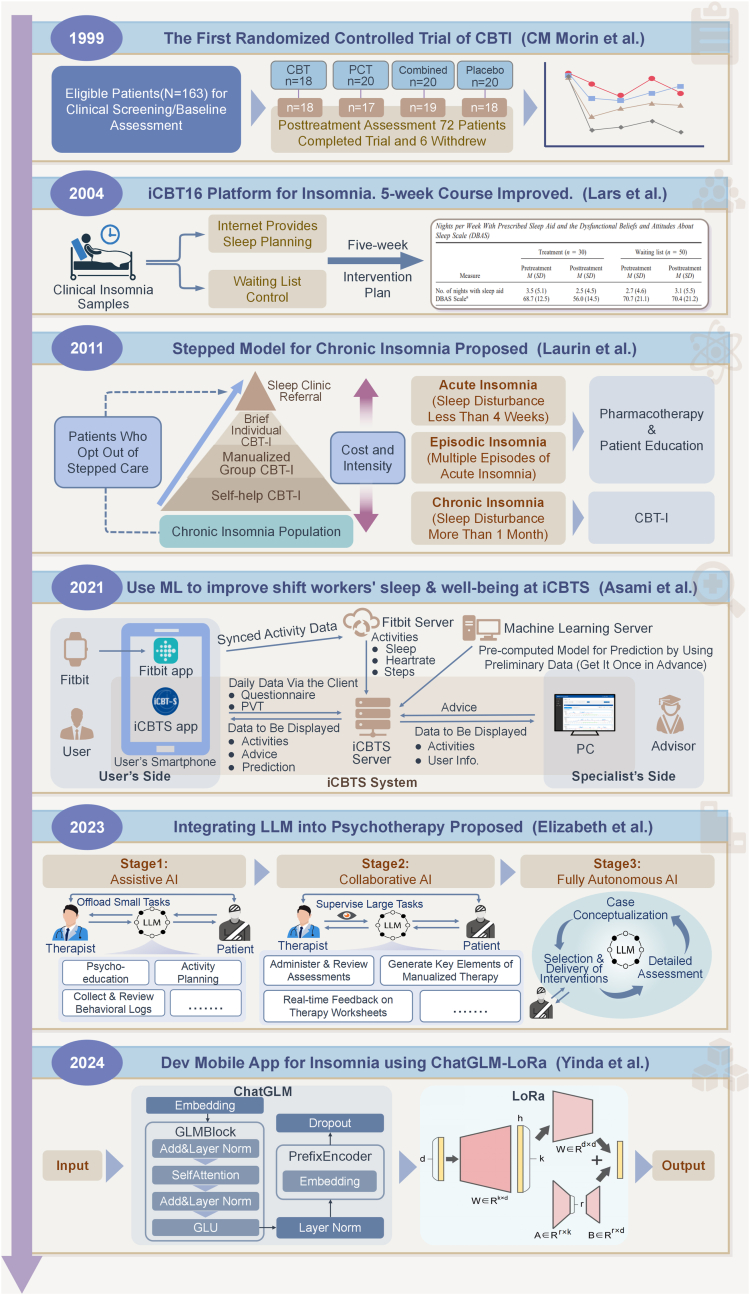
The historical development of important breakthroughs in CBTI. This timeline highlights key studies and advancements in the field, from the first randomized controlled trial of CBTI in 1999 (CM Morin et al.) to the development of mobile AI platforms for insomnia treatment in 2024 (Yinda et al.). The timeline also showcases various milestones, such as the introduction of stepped care models (Laurin et al., 2011), improvements in internet-based CBT (Lars et al., 2004), and the integration of machine learning in CBTI (Asami et al., 2021), with a focus on AI-assisted and autonomous models in recent years (Elizabeth et al., 2023).

The advent of digital health technology and AI has accelerated the transition from structured eCBT-I to AI-driven CBT-I [[50], [51], [52]]. This is not just a technological innovation, but an attempt to fundamentally redefine the healthcare delivery model [53]. The integration of AI enables customized treatment regimens, real-time monitoring of sleep patterns, and adaptive adjustments based on data analysis [54]. This approach aims to address the limitations of conventional treatment methods through the utilization of intelligent methodologies [[55], [56], [57]]. The historical development of important breakthroughs in cbti is charted in Fig. 1.

With the digital transformation of CBT-I, eCBT-I as a modular, internet-based program has expanded access and scalability [45]. However, its reliance on fixed content and limited interactivity reduces responsiveness to individual needs. The absence of real-time feedback mechanisms further limits timely intervention and can compromise treatment outcomes. Concurrently, the limited user interactivity and personalized support inherent in eCBT-I also diminish patient engagement and compliance [58].

### 3.2 Advantages and effects of AI application in CBT-I

**Fig. 2:**
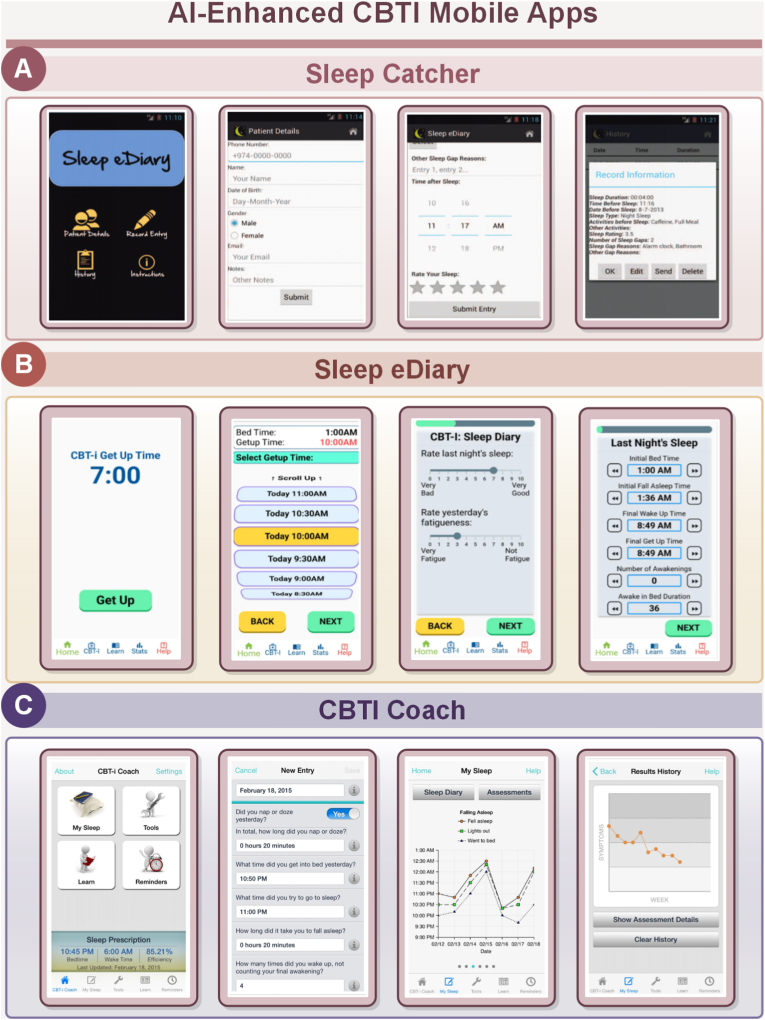
AI enhanced CBTI mobile apps. Panel A displays Sleep Catcher, which collects and records patient details, and allows users to input sleep-related data. Panel B showcases Sleep eDiary, which helps users track their sleep patterns, set wake-up times, and rate sleep quality. Panel C presents CBTI Coach, offering personalized sleep prescriptions, tracking of sleep progress, and providing results history to help improve cognitive behavioral therapy for insomnia (CBTI) outcomes. These AI-enhanced applications aim to optimize the CBTI process for users.

In the face of the above challenges, the in-depth integration of AI technology and medical research provides new solutions for optimizing CBT-I [[59], [60], [61]]. AI-driven CBT-I not only inherits the advantages of eCBT-I in expanding treatment coverage, but also overcomes the limitations of traditional eCBT through intelligent means. By leveraging advanced algorithms and big data, AI-driven systems can adjust treatment in real time, deliver personalized guidance, and adapt to each patient's evolving condition. Fig. 2 shows some of the mobile applications that utilize AI to enhance CBTI implementation. In particular, the application of social robots and large language models (LLMs) further enhances the quality of human-computer interaction, improves patient engagement and compliance, and facilitates the efficiency and professional judgment of mental health professionals [[62], [63], [64]]. Consequently, AI-driven CBT-I represents a significant step toward personalized medicine. Table 1 outlines the AI tools currently applied in CBT-I interventions.

**Table 1:** AI tools used in current CBTI intervention.

AI tools	Description
Sleepio [66]	Sleepio is a CBTI-based digital sleep treatment tool that uses artificial intelligence to analyze users' sleep data and provide personalized sleep interventions based on user feedback. AI adjusts the treatment content according to the user's sleep pattern, behavioral habits and emotional state to help users improve their sleep quality.
Shuti [67]	Shuti is a sleep intervention tool developed by Microsoft that combines CBTI and AI technology to provide users with personalized sleep treatment recommendations by analyzing their sleep patterns. It aims to optimize the treatment process through intelligent algorithms to improve insomnia symptoms.
CBT-i Coach [68]	CBT-i Coach is a sleep treatment app jointly developed by the United States Department of Veterans Affairs and the National Institutes of Health to help users manage insomnia on their own. It integrates CBTI principles and provides personalized treatment plans by collecting users' sleep logs and mood data.
Somryst [69]	Somryst is an FDA-approved digital therapeutic tool developed by Pear Therapeutics that is specifically designed to treat insomnia. Combining CBTI technology and AI, Somryst helps users improve their sleep by analyzing their sleep data and behavioral patterns and adjusting treatment strategies in real time.
Headspace [70]	Headspace for Sleep is a part of the Headspace app that combines meditation and CBTI technology to provide users with personalized sleep improvement recommendations through AI analysis of their sleep data, meditation feedback, and emotional states.
Pzizz [71]	Pzizz is a sleep aid app that combines CBTI and AI, using personalized audio therapy to help users fall asleep. AI generates customized audio content based on the user's sleep data to improve sleep quality.
Insomnia Coach [72]	Insomnia Coach is an app developed by the U.S. Department of Veterans Affairs and designed specifically for CBTI interventions. It helps users improve their sleep habits through intelligent feedback and provides customized adjustments to their sleep patterns.
RescueTime [73]	RescueTime is a time management tool that uses AI to analyze users' daily activities, including sleep patterns. While it is not a dedicated CBTI tool, it can indirectly help improve sleep by tracking sleep behavior and lifestyle data。
SleepScore [74]	SleepScore is a sleep health app that combines CBTI principles and AI analytics to help users improve their sleep quality. By tracking sleep patterns and behavioral data, SleepScore provides personalized sleep improvement recommendations.
Calm [75]	Calm is a meditation and sleep app that combines CBTI technology and uses AI to analyze users' sleep data, mood swings, and meditation feedback. It provides personalized meditation training based on the individual needs of users to help improve sleep quality and reduce insomnia symptoms.
	

The social robot offers a new way of implementing CBT therapy by simulating human conversation and emotional interaction. It not only provides structured support, but also accompanies patients at all times via the Internet and mobile devices, enhancing accessibility and flexibility of treatment. Studies have shown that social robots can directly interact with patients, promoting engagement and adherence, thus improving treatment outcomes. For example, in 2019, Francesca Dino et al. used Ryan, a social robot, to deliver internet-delivered cognitive behavioral therapy (iCBT) to older adults with mild-to-moderate depression, evaluating the effectiveness of the robot-based iCBT and the user's liking for the approach through the results of participants' quantitative analyses of their verbal responses (e.g., word count statistics and sentiment analyses), facial scale mood scores, and exit surveys. The results suggest that robot-assisted therapy is an effective alternative to traditional human treatment [65]. In 2021, Sooah Jang et al. tested the “Todaki” chatbot with patients experiencing attention deficits and reported significantly fewer symptoms compared to a book-based control. Patients used the chatbot on average 20 times over four weeks, indicating sustained engagement. However, chatbots also face limitations, such as restricted conversational fluency and limited understanding of complex emotions, which may affect therapeutic experience [62].

In addition to interacting directly with patients, social robots can be used as an aid to psychotherapists, facilitating data-driven and adaptive approaches to help manage patient progress more efficiently [76]. In 2022, Torrey A. Creed created AI software capable of processing and analyzing audio recordings of CBT therapy sessions—Project AFFECT. This system not only identifies and evaluates the presence and quality of key CBT elements, such as whether therapists are applying techniques correctly and according to established standards, but also delivers structured feedback in real time to help therapists refine their practice and better meet patient needs [63]. In 2023, Anja Thieme et al. successfully developed an AI application that predicts the treatment outcomes of patients who receive human-supported iCBT for depression and anxiety disorders, in particular, demonstrating how AI technology can be integrated into specific clinical settings, thereby improving iCBT support treatment outcomes and service quality. At the same time, the study also emphasized the importance of responsible design and deployment of AI to ensure that technological advances truly benefit patients and supporters on a daily basis without negatively impacting them [77]. In 2024, Jeremy J. Coleman used the Lyssn system to parse and identify the specific elements of computer-assisted cognitive behavioral therapy (CACBT) that were most effective, finding that guided discovery, understanding, and interpersonal efficacy were the most effective in alleviating anxiety, and that the proportion of open-ended questions posed was the most influential in the outcome of treatment. This study demonstrates the potential of utilizing the power of AI to dig deeper and understand the core mechanisms of CACBT, providing technical support to improve the overall effectiveness of treatment and patient recovery rates [78]. Although these examples are not strictly social robots, they demonstrate how AI technologies can augment robotic systems in CBT by enhancing assessment and feedback mechanisms.

Social robotics-enabled CBT therapies have evolved from early systems that provided structured guidance, such as the Ryan robot, to advanced AI systems that can handle complex conversations, provide immediate feedback, and predict treatment outcomes and optimize strategies. Although the literature on direct application to CBT-I is limited, research from other CBT fields suggests that social robots have great potential for application in CBT-I therapy.

### 3.3 Challenges and future directions

In addition, social robot-assisted CBT therapy faces a number of challenges that CBT-I therapy will face. First, social robots rely on preset rules and scripts, which means that their responses tend to be fixed and lack flexibility and personalization [79]. Second, social robots may have difficulty dealing with complex or unintended situations because they cannot respond as flexibly as human psychotherapists [80]. In addition, their limited capacity for learning prevents deeper adjustments based on individual feedback [81]. These limitations make their effectiveness and applicability challenging in treatment contexts that require a high degree of individualization, emotional sensitivity, and real-time adaptability.

## 4 Future directions: applications and effectiveness of LLMs in CBT-I

### 4.1 Applications of LLMs in CBT-I

**Fig. 3:**
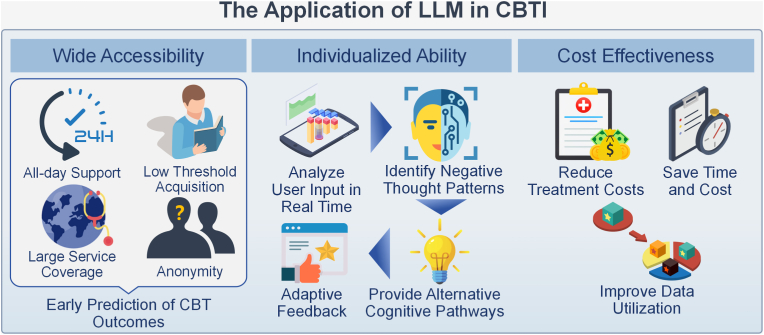
The application of LLM in CBTI. The diagram highlights three key areas: Wide Accessibility, Individualized Ability, and Cost Effectiveness. Under Wide Accessibility, LLM offers all-day support, low threshold acquisition, large service coverage, anonymity, and early prediction of CBT outcomes. In Individualized Ability, LLM can analyze user input in real time, identify negative thought patterns, provide adaptive feedback, and offer alternative cognitive pathways. Finally, under Cost Effectiveness, LLM helps reduce treatment costs, save time and cost, and improve data utilization.

With advances in deep learning and computational power, LLMs have emerged as a promising tool to extend the reach of CBT-I. Built through large-scale pre-training and transfer learning, they generate natural and context-sensitive dialogue, offering users personalized support that goes beyond the limitations of earlier digital tools such as social robots. By capturing complex linguistic structures and semantics, LLMs can assist CBT-I delivery in several ways: providing rapid access to psychoeducational content, offering personalized advice, reducing therapist workload, enhancing user engagement, and supporting clinician training and education. Fig. 3 illustrates three key dimensions of these applications: wide accessibility, individualized capability, and cost-effectiveness. To provide a structured overview, we summarize representative 10.13039/100014258LLM use-cases, supporting studies, limitations, and safeguards in Table 2.

**Table 2:** Representative LLM-based applications relevant to CBT-I.

LLM Use-Case	Descriptions	Key Limitations	Safeguards Future Needs
Psychoeducation and Q&A	LLMs answer insomnia-related questions, reframe maladaptive thoughts, or explain CBT-I principles	Risk of hallucinations, shallow reasoning	Grounding responses in validated sources; explicit disclosure of non-clinical status
CBT-style Dialogue Delivery	Fine-tuned LLMs generate CBT-consistent therapeutic exchanges with empathy and structure	Risk of agenda drift, limited depth	Structured prompts, clinician supervision, fidelity checks
Patient Simulation for Training	LLMs simulate diverse patient scenarios for therapist training and benchmarking	Not equivalent to lived patient experience	Rubric-based evaluation; expert oversight
Risk Monitoring Decision Support	LLMs flag risk events (e.g., suicidality, non-adherence) and suggest next steps	Workflow fit, automation bias	Human-in-the-loop review; audit logs
Fidelity Feedback	LLMs evaluate session transcripts and provide feedback on CBT-I fidelity	Domain shift, privacy concerns	Secure pipelines; patient consent

Nathan Hodson et al. explored the cognitive biases in OpenAI's ChatGPT-4 and Google's Bard's understanding of CBT psychotherapists' ideas, and showed that these LLMs can provide reasonable suggestions for identifying and reconstructing unhelpful ideas, but should not be relied upon as primary therapeutic guides, underscoring both their promise and limitations [82]. In 2024, Mian Zhang et al. constructed the CBT-BENCH assessment benchmark, which comprehensively assessed the performance of LLMs in CBT tasks, i.e., from simple memory-based knowledge reproduction to complex cognitive structure analysis and effective therapeutic dialogue generation. The results show that while LLMs performed well in knowledge recall and multiple-choice questions, they struggled with real-world scenarios requiring nuanced analysis of patients' cognitive structures and effective therapeutic responses [83]. In the same year, Talha Tahir et al.'s study focused on enhancing the performance of LLMs through fine-tuning based on CBT theory. The CBT-fine-tuned models significantly outperformed instruction-tuned baselines, with an average improvement of 11.33 points in CTRS scores (p < 0.001), with Llama 3.1 8b achieving the best performance. These models effectively implemented core CBT techniques and produced empathetic responses, though limitations persisted in agenda adherence, depth of exploration, and long-context coherence [84]. This research offers significant insights into the application of AI techniques to expand the scope of CBT services and highlights future directions and considerations [85]. In a study by Nathan Hodson, Mian Zhang, and Talha Tahir, the use of LLMs in CBT has evolved from theoretical evaluation to performance optimization, demonstrating their capacity for basic knowledge reproduction, core CBT technology implementation, and empathetic response.

However, the extant literature has primarily centered on the model's capabilities and technical enhancements, with scant attention devoted to the implementation of these capabilities in real-world training settings. Ruiyi Wang et al. have developed a novel patient simulation framework, PATIENT-Ψ, for CBT training. Utilizing LLMs to simulate real patient interactions, the researchers demonstrated that PATIENT-Ψ-TRAINER significantly enhances trainees' perceptual skill levels and self-confidence, surpassing the effects of traditional training methods such as textbooks, video learning, and non-patient role-playing. Experts concluded that PATIENT-Ψ is closer to real patient interactions than GPT-4 and shows great potential to enhance trainees' professional competence. These findings emphasize that LLMs can not only augment engagement and training quality but also bridge the gap between theory and practice in CBT education.

### 4.2 Applications and challenges of LLMs in CBT-I

The above research demonstrates the significant advantages of LLMs in CBT-I application. First, LLMs can help mitigate the shortage of psychotherapists by providing round-the-clock support through online platforms, broadening access to mental health services in underserved regions [86]. Second, LLMs' personalization capability is also an important feature. By analyzing user input in real time, it can generate highly relevant suggestions tailored to individual needs, such as identifying negative thought patterns and providing alternative cognitive pathways to enhance the user's intervention experience [87]. In addition, LLMs excel in terms of cost-effectiveness. Compared to expensive and time-consuming traditional treatments, LLMs offer a more cost-effective and efficient solution for mental health support, helping to reduce the pressure on the mental health delivery system [82].

**Fig. 4:**
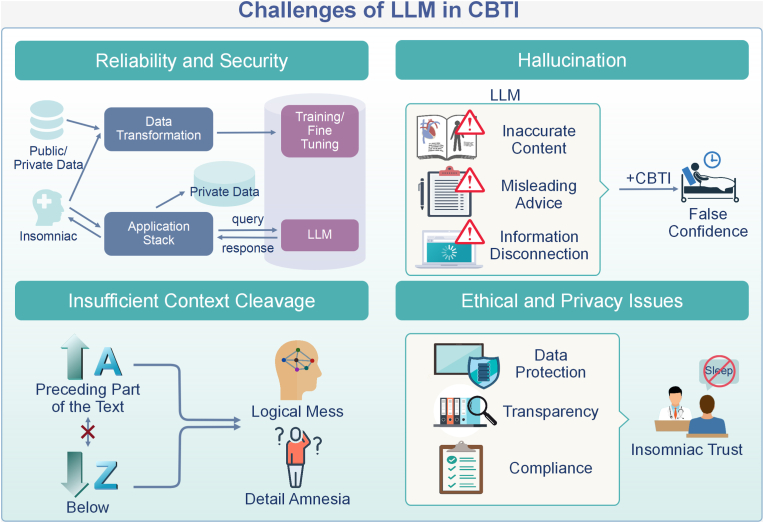
Challenges of LLM in CBTI. The diagram highlights four main areas: Reliability and Security, Hallucination, Insufficient Context Cleavage, and Ethical and Privacy Issues. Each area details specific problems such as data transformation, training/fine-tuning leading to inaccurate content, misleading advice, and privacy concerns.

Despite the compelling potential of LLMs in CBT-I, it still faces many challenges in its practical application. First, current research has focused on the model's capabilities and technological improvements, and there is a lack of insight into how these capabilities can be effectively integrated into real-world training settings. Second, the issue of model credibility and security has attracted attention [88]. LLMs may generate hallucinations—producing inaccurate or misleading information—which could negatively affect users' mental health. Ethical and privacy issues are equally critical [89]; safeguarding sensitive health data requires robust confidentiality and compliance frameworks. In addition, limited contextual understanding and weak long-dialogue coherence hinder their ability to sustain in-depth CBT-I sessions, which can reduce both trust and treatment effectivenes [88,90]. There is still room for further exploration on how to evaluate the effectiveness of this new training modality, especially in terms of long-term effects and applicability in different cultural contexts. Fig. 4 shows the challenges of LLM in CBTI.

In the future, to address the current challenges in the application of LLMs in CBT-I, its effectiveness and reliability can be enhanced through further optimization and integration of technologies. For example, the introduction of multimodal data to support the contextual comprehension ability of LLMs or the enhancement of its long-term dialogue coherence through fine-tuning techniques can improve the user experience. Concurrently, it is imperative for developers and ethicists to collaborate in the formulation of a rigorous privacy protection framework. This collaborative effort is crucial for ensuring that the applications of LLMs in the domain of mental health are safe, secure, and trustworthy. The implementation of these enhancements will facilitate the expansion of LLMs applications in CBT-I.

### 4.3 Future prospects of CBT-I with LLMs

In order to achieve these goals more effectively, integrating LLMs with complementary mental health technologies is essential to enhancing their practical value. By combining emotion recognition sensors, behavioral tracking tools, and wearable devices, comprehensive monitoring of the user's state can be achieved. For example, Massimiliano de Zambotti et al. evaluated the performance of a multisensor sleep tracker and polysomnography in measuring sleep and its staging, and by integrating sleep monitoring data and user behavioral analytics, LLMs were able to generate more precise and personalized treatment recommendations [91]. For patients who have difficulty falling asleep, the system can provide targeted mood regulation exercises or relaxation training based on real-time sleep patterns [92]. Yuhong Zhang et al. combined LLMs, EEG, and eye-gaze technologies to achieve word-level neurological state classification, demonstrating that multimodal data can accurately capture cognitive states. This multimodal fusion not only strengthens 10.13039/100014258LLM functionality but also improves patient acceptance and adherence to interventions, offering more effective support for mental health care [93].

Second, the application of LLMs in CBT-I requires deep interdisciplinary collaboration. Psychologists can ensure clinical validity by providing therapeutic frameworks and scientific validation; computer scientists can focus on model optimization and dialogue quality; and ethicists can design responsible data-processing and interaction frameworks. For example, Woebot® (WB001), an agent-guided cognitive behavioral therapy software for postpartum depression treatment, combines human-computer interaction design, machine learning techniques, and principles of CBT and interpersonal therapy [94]. Through multidisciplinary collaboration, the technical and social challenges that LLMs may face in mental health applications can be addressed more comprehensively to ensure their feasibility and safety in real-world scenarios [95].

**Fig. 5:**
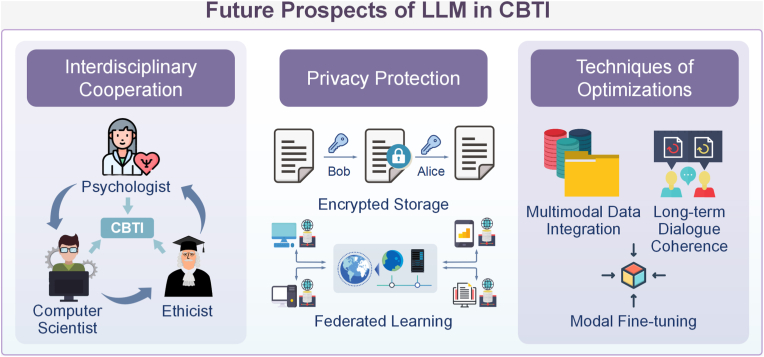
Future prospects of LLM in CBTI. The diagram is divided into three key areas: interdisciplinary cooperation, privacy protection, and optimization techniques. In terms of interdisciplinary cooperation, psychologists, computer scientists, and ethicists work together to advance CBTI. Regarding privacy protection, the use of encrypted storage and federated learning is emphasized to ensure data security and privacy. For optimization techniques, it discusses methods such as multimodal data integration, long-term dialogue coherence, and model fine-tuning to enhance the effectiveness and application of LLM in CBTI.

In addition, privacy protection and ethical compliance remain critical priorities when handling sensitive mental health data. A strict data protection framework is needed, including encrypted storage of data, transmission security, and access control. At the same time, transparency of data use should be clarified to inform users of the functions, limitations, and potential risks of LLMs to avoid adverse consequences due to over-reliance [96]. Design strategies such as federated learning can reduce reliance on centralized storage, while clear exit mechanisms and crisis management protocols should ensure patients can access human intervention when necessary. Fig. 5 illustrates these future directions.

The application of LLMs in CBT-I shows great promise, but improvements are still needed to address its limitations. Through continued optimization of the technology, enhanced interdisciplinary collaboration, and improved privacy protection mechanisms, LLMs are expected to become a more efficient and trustworthy mental health intervention tool, providing broader and personalized support for insomnia patients. Continuous exploration in this area will bring new possibilities for the popularization and enhancement of mental health services.

## 5 Conclusion

This review analyzes the current status, challenges, and future directions of cognitive behavioral therapy (CBT-I) and its Internet version (eCBT-I), focusing on the potential of artificial intelligence (AI), especially large language models (LLM), in these therapies. First, the basic principles and therapeutic effects of CBT-I are introduced, and the challenges faced by traditional CBT-I such as low acceptance and long treatment cycles are discussed. Then, the advantages of eCBT-I in flexibility, accessibility and cost-effectiveness are analyzed. We also explore how AI and LLM can improve outcomes by personalizing treatment and enhancing patient interaction, and raise privacy and ethical issues. To sum up, AI-enhanced CBT-I has great potential, and future research will promote its wider application and improve the effectiveness of insomnia treatment.

## CRediT authorship contribution statement

**Chenxing Lin:** Writing – original draft, Investigation, Data curation. **Chen Cheng:** Writing – original draft, Investigation, Data curation. **Yu Xia:** Writing – original draft, Investigation, Data curation. **Ruoyun Wang:** Visualization, Methodology, Investigation. **Luying Zhang:** Visualization, Methodology, Investigation. **Lintong Han:** Visualization, Methodology, Investigation. **Darong Hai:** Visualization, Methodology, Investigation. **Chenyue Dai:** Visualization, Methodology, Investigation. **Tianen Dai:** Visualization, Methodology, Investigation. **Luhuan Cao:** Visualization, Methodology, Investigation. **Xingyu Zhu:** Visualization, Methodology, Investigation. **Hao Chen:** Visualization, Methodology, Investigation. **Li Zeng:** Visualization, Methodology, Investigation. **Chufan Ren:** Visualization, Methodology, Investigation. **Dongren Yang:** Visualization, Methodology, Investigation. **Yijun Wang:** Visualization, Methodology, Investigation. **Meijia Zhang:** Visualization, Methodology, Investigation. **Zefei Mo:** Visualization, Methodology, Investigation. **Yinda Chen:** Visualization, Methodology, Investigation. **Jianwei Shuai:** Writing – review & editing, Supervision, Project administration, Funding acquisition, Conceptualization. **Rongwen Yu:** Writing – review & editing, Supervision, Project administration, Funding acquisition, Conceptualization. **Yuming Peng:** Writing – review & editing, Supervision, Project administration, Funding acquisition, Conceptualization.

## Data availability statement

Not applicable.

## Declaration of competing interest

The authors declare that the research was conducted in the absence of any commercial or financial relationships that could be construed as potential conflicts of interest.
